# Numerical Simulation of Physical Fields during Spark Plasma Sintering of Boron Carbide

**DOI:** 10.3390/ma16113967

**Published:** 2023-05-25

**Authors:** Song Zhang, Wei Liu, Weimin Wang, Ying Gao, Aiyang Wang, Qianglong He, Wenhui Bai, Runfeng Li

**Affiliations:** 1 State Key Laboratory of Advanced Technology for Materials Synthesis and Processing, Wuhan University of Technology, Wuhan 430070, China; 2 School of Materials Science and Engineering, Wuhan University of Technology, Wuhan 430070, China

**Keywords:** boron carbide, spark plasma sintering, numerical simulation, temperature distribution, relative density distribution

## Abstract

Spark plasma sintering is a new technology for preparing ceramic materials. In this article, a thermal-electric-mechanical coupled model is used to simulate the spark plasma sintering process of boron carbide. The solution of the thermal-electric part was based on the charge conservation equation and the energy conservation equation. A phenomenological constitutive model (Drucker-Prager Cap model) was used to simulate the densification process of boron carbide powder. To reflect the influence of temperature on sintering performance, the model parameters were set as functions of temperature. Spark plasma sintering experiments were conducted at four temperatures: 1500 °C, 1600 °C, 1700 °C, and 1800 °C, and the sintering curves were obtained. The parameter optimization software was integrated with the finite element analysis software, and the model parameters at different temperatures were obtained through the parameter inverse identification method by minimizing the difference between the experimental displacement curve and the simulated displacement curve. The Drucker-Prager Cap model was then incorporated into the coupled finite element framework to analyze the changes of various physical fields of the system over time during the sintering process.

## 1. Introduction

Boron carbide is a material suitable for a variety of high-performance applications due to its excellent properties such as high hardness (30 GPa), low density (2.52 g/cm^3^), high melting point (2450 °C), high Young’s modulus (448 GPa), chemical inertness, high neutron absorption cross section (600 barns), excellent thermoelectric properties, etc. [1,2,3,4,5]. This combination of properties makes boron carbide useful in a wide range of industrial applications, including armor systems, nuclear power plants, hard metal tools, automobiles, and even biomedicine. However, the consolidation of B_4_C is very complicated due to its high melting point and low self-diffusion coefficient. Densification requires very high sintering temperatures due to the presence of predominantly covalent bonds in B_4_C [4]. Densification of boron carbide can be achieved without reducing its mechanical properties by using suitable sintering aids and/or applying external pressure (e.g., hot pressing, hot isostatic pressing). SPS technology helps achieve dense products without any coarsening of the grains [2,6,7,8].

The SPS process is a complex physical phenomenon that involves multi-field coupling effects of the electric field and temperature field, as well as the densification process of powders. It is initiated by Joule heating caused by the current, which leads to a rapid heating rate and large temperature gradients within the sintered sample. Numerical simulation is an effective method for understanding the mechanism of SPS and capturing the gradients of various physical fields in the final sample [9,10,11,12,13,14,15,16,17,18]. 

Achenani et al. [15] used the finite element model to simulate the temperature distribution in the sintering process of alumina by SPS, and its parameters were adjusted to reproduce the measured temperature values. In particular, the axial asymmetry of the system was taken into account by considering the asymmetric thermal boundary conditions. In addition to the radial temperature gradient, the axial temperature gradient also appeared in the sample, and the axial temperature distribution was asymmetric. However, the sample was considered to be completely dense without considering the densification process and mechanical properties of the sample.

Mohammad et al. [11] conducted a numerical simulation study on spark plasma sintering of TiC samples. The governing equations, including heat conduction and current distribution, were solved with a finite element method using COMSOL software. The densification and mechanical properties of the sample were not considered. Sakkaki et al. [10] performed numerical simulation of the temperature and current distribution in the sintering process of ZrB_2_ and used a finite element method to solve the current density equation and energy conservation equation. However, again, the sample was treated as a dense sample, and the densification process and mechanical properties were not considered.

Current works mostly focus on temperature and electric fields but consider samples to be completely dense. In fact, the mechanical properties of the material and the densification process must be considered to obtain accurate temperature and stress fields in the sample.

Models of different scales have been used to describe the densification behavior of powders. Engelke et al. [19] took into account the complexity and variability of particle shape and represented each particle as a truncated sphere. They used the discrete element method to simulate the multi-dispersed filler with up to 16,000 particles and reproduced the shrinkage and grain coarsening during the sintering process. So et al. [20] proposed a discrete element method to simulate the elastic, plastic, and viscoelastic deformation of all-solid-state battery particles. Matsuda [21] proposed a discrete element model capable of handling temperature changes to simulate the sintering process, and used the numerical model established to illustrate the influence of surface diffusion on shrinkage behavior. Although the discrete element method can explain the densification behavior of powders at the microscopic level, it requires a lot of computational power and is difficult to couple with the thermal-electrical finite element analysis. Therefore, sintering models based on the general continuum theory are more widely used. Shi et al. [22] used Skorohod and Olevsky viscous sintering constitutive relations to simulate the shrinkage and relative density variation of ceramics during sintering. Mani’ere et al. [23] use the SOVS (Skorohod-Olevsky Viscous Sintering) model to conduct finite element modeling analysis of a printed cup and predict the sintering shrinkage of samples with complex geometric shapes. Kakanuru et al. [24] implemented the SOVS model as a custom creep model in finite element software, and determined the required parameters of the SOVS model by minimizing the error between the experimental and simulated relative densities. The dimensional variation of additive-manufactured green bodies during sintering was analyzed. Different phenomenological models have also been introduced for sintering in the SPS process. Wang et al. [25] established a coupled electrical-thermal-mechanical model to analyze the temperature and stress distribution of the SPS process. The densification process of powder was realized by applying the actual displacement curve, but only the linear elastic deformation of powder was considered. Mondalek et al. [26] established a three-dimensional finite element simulation to analyze the current, temperature, and pore distribution, and discussed the effects of the physical properties of the powder and the geometry of the mold on the current and temperature distribution. The powder densification process was simulated using the Abouaf constitutive model. Wolff et al. [16] established a coupled electrical-thermal-mechanical model in ABAQUS software to simulate the sintering densification process of nickel powder, a modified micromechanical model of porous material was used to describe the densification behavior of the material, and two SPS experiments were used to determine the parameters of the constitutive equation. Two additional SPS experiments were used to verify the accuracy of the model. The results showed that the prediction of the model was in good agreement with the experimental densification data. Ni et al. [27] simulated the dry pressing and solid-state sintering processes of MgTiO3 ceramic powder using the Drucker-Prager Cap model for the dry pressing part, the model parameters were determined experimentally at different relative densities, and the solid-state sintering part was simulated using the SOVS model. The feasibility of the method to simulate the densification process was confirmed by comparing the simulation results of complex shapes with the experimental results. Bai et al. [28] used the Drucker-Prager Cap model, which could accurately describe the densification behavior of powder materials, to perform numerical analysis on the spark plasma sintering process of boron carbide powder. The parameters of the Drucker-Prager Cap model were identified at different temperatures based on spark plasma sintering experiments. Electrical-thermal-mechanical finite element simulation of spark plasma sintering of boron carbide powder was performed at 1750 °C and 1850 °C. Temperature, stress, and relative density fields were studied numerically. By comparing the model results with experimentally measured temperature and relative density, the continuum finite element method using the Drucker-Prager Cap model was verified.

In previous work, the densification process was only considered at a single temperature. However, according to previous computational results of the temperature field within the sample, significant temperature gradients exist, resulting in significant variations in sintering performance in different regions [10,11,25,29,30,31]. Additionally, the sintering process of the powder was modeled with an elastic constitutive equation [25,31], or separating plasticity and creep modeling, which increased the complexity of the simulations [27].

In this study, the DPC model was used to simulate the sintering process of boron carbide ceramics at high temperatures. The plasticity and creep parameters of the DPC model were obtained at different temperatures through the parameter inverse identification method. A finite element model of multi-physics coupling was established to simulate the spark plasma sintering process of boron carbide ceramics. The current density distribution, temperature distribution, stress field distribution, and relative density distribution inside the sample were calculated for the final system. By utilizing the established finite element model, an investigation was conducted on the relationship between nominal temperature and actual temperature at different measurement points, as well as the impact of mold dimensions on the thermal distribution of the sample.

## 2. The Drucker-Prager Cap Model and Parameter Acquisition

### 2.1. The Drucker-Prager Cap Model

**Figure 1:**
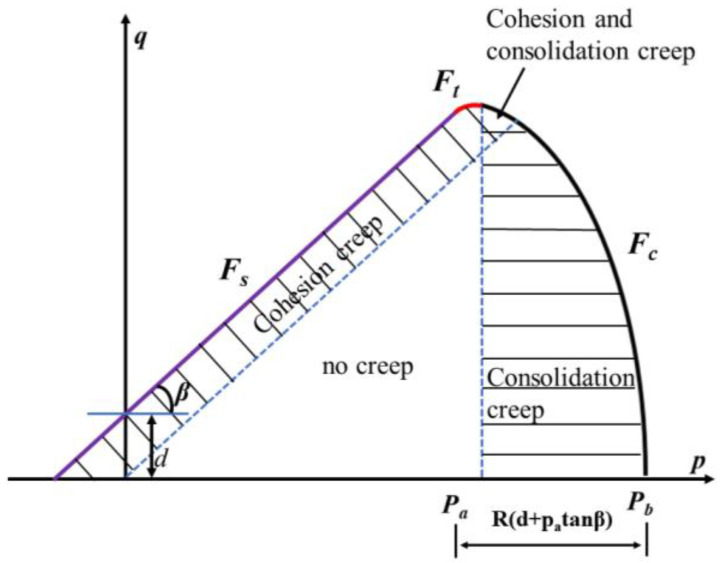
DPC model with creep mechanisms in the *p*-*q* plane.

The extended DPC model, as shown in Figure 1, defines the yield surface in the p−q coordinate system, which consists of three curves: the shear failure surface $F_{s}$, the transition zone $F_{t}$, and the cap yield surface $F_{c}$. They are expressed as:

(1) $$F_{s}=q-ptan\beta -d=0$$

(2) $$F_{c}=\sqrt{{\left(p-p_{a}\right)}^{2}+{\left[\frac{Rt}{\left(1+\alpha -\alpha /\cos\beta \right)}\right]}^{2}}-R(d+p_{a}\tan\beta )=0$$

(3) $$F_{t}=\sqrt{{\left(p-p_{a}\right)}^{2}+{\left[t-(1-\frac{\alpha }{\cos\beta })(d+p_{a}\tan\beta )\right]}^{2}}-\alpha (d+p_{a}\tan\beta )=0$$

where p is the hydrostatic pressure stress, q is the Mises equivalent stress, d is the cohesive strength, β is the friction angle, R is the cap eccentricity, $p_{a}$ is the evolution parameter, $p_{b}$ is the static hydrostatic pressure yield stress, and α is a small constant used to define the smooth transition surface between $F_{s}$ and $F_{c}$. Specifically, α = 0 is required for the creep analysis of ABAQUS. The hardening/softening rule is a user-defined function related to hydrostatic compression yield $P_{b}$ and volumetric inelastic (plastic and/or creep) strain $P_{b}=P_{b}(\varepsilon^{pl},\varepsilon^{cr})$.

In order to consider the creep behavior in the simulation, a cap creep model was used. The creep model is related to the Drucker-Prager Cap model plasticity. Depending on the loading type, two creep mechanisms are active: cohesive creep and consolidation creep. The cohesive creep mechanism follows the active plastic type in the shear failure plastic zone, and the consolidation creep mechanism follows the active plastic type in the cap zone. As shown in Figure 1, there is a region where both mechanisms are active. For cohesion creep mechanism and the given stress state, the equivalent creep stress ${\overset{-}{\sigma }}^{cr}$ calculation formula is:

(4) $${\overset{-}{\sigma }}^{cr}=\frac{q-p\tan\beta }{1-\tan\beta /3}$$

When using the consolidation creep mechanism, the stress is replaced by the effective creep stress ${\overset{-}{p}}^{cr}=p-p_{a}$. In the process of die compression, the consolidation creep mechanism is dominant [32]. Additionally, the shape of the cap has a significant impact on the mechanism and magnitude of creep deformation [33].

There are two power models for the creep behavior, which are the power law of time hardening and the power law of strain hardening, respectively. The time hardening law is more applicable in this study when the stress state is held constant during analysis (Abaqus^®^: Theory Manual and analysis user’s Manual). The expression is:

(5) $${\dot{\overset{-}{\varepsilon }}}^{cr}=A{\left({\overset{-}{\sigma }}^{cr}\right)}^{n}t^{m}$$

where t represents the time and ${\dot{\overset{-}{\varepsilon }}}^{cr}$ is the equivalent creep strain rate. Under the consolidation creep mechanism, the equivalent creep stress ${\overset{-}{\sigma }}^{cr}$ is replaced with the effective creep stress ${\overset{-}{p}}^{cr}$. The parameters A, n, and m depend on the material.

The total strain increment can be written as:

(6) $$d\varepsilon =d\varepsilon^{el}+d\varepsilon^{pl}+d\varepsilon^{cr}$$

where dε is the total strain rate, $d\varepsilon^{el}$ is the elastic strain rate, $d\varepsilon^{pl}$ is the inelastic (plastic) time-independent strain rate, and $d\varepsilon^{cr}$ is the inelastic (creep) time-dependent strain rate.

The volumetric plastic strain, as an internal variable on which the control parameter depends, is related to the relative density by the following formula [34,35]:

(7) $$\varepsilon_{v}^{p}=\ln\left(\frac{\rho }{\rho_{0}}\right)$$

where ρ and $\rho_{0}$ represent the current and initial (i.e., corresponding to zero plastic strain) relative density. The relationship between relative density and strain is realized by the USDFLD subroutine in ABAQUS.

### 2.2. Parameter Identification of Boron Carbide Powder

**Figure 2:**
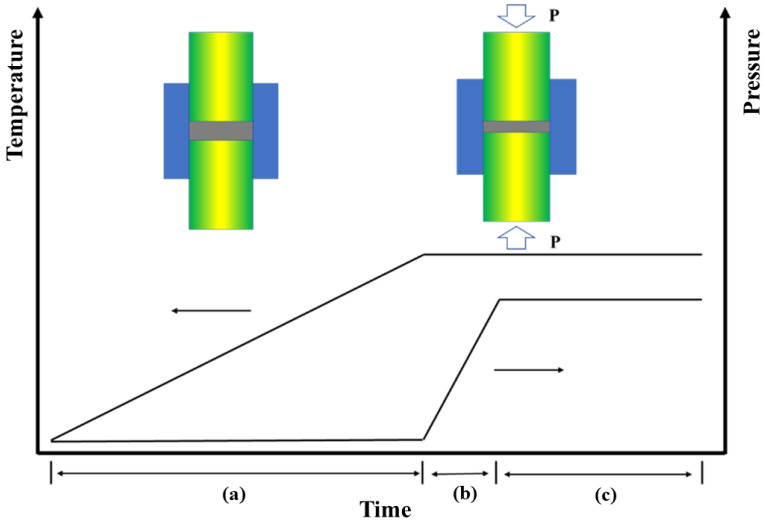
Schematic illustrations of sintering process. (a) Heating up. (b) Loading pressure. (c) Soaking time.

The B_4_C ceramics were prepared using the SPS system (FCT, GmbH, Effelder-Rauenstein, Germany). The raw B_4_C powder (40 g) was loaded into a graphite die with an internal diameter of 50 mm and placed in the SPS chamber under vacuum conditions. The powder was pre-compacted at room temperature at 16 kN before sintering and then consolidated at 1500 °C, 1600 °C, 1700 °C, and 1800 °C, respectively. The sintering process involved three stages: (a) The powder was heated up to the specified temperature at a rate of 100 °C/min under pre-pressure. (b) The pressure was increased to 30 MPa within 2 min at a constant temperature. (c) The sample was held at constant temperature and pressure for 5 min. The lower punch moved upward and the upper punch was fixed. The temperature and pressure evolution over time are presented in Figure 2. The displacement-time curves were recorded by the FCT system.

For the DPC model, the parameters E, ν determine the elastic properties; β, d, R, and $P_{b}$ determine the cap plasticity; and A, n, and m determine the creep behavior of the material. These parameters were set as functions of relative density to describe the densification behavior of powders more accurately [34,35,36,37]. The instantaneous relative density was calculated as follows:

(8) $$\rho_{RD}=\frac{L_{f}}{L}\rho_{f}$$

where $\rho_{RD}$ is the instantaneous relative density, $\rho_{f}$ is the final relative density of the sample, L is the instantaneous height, and $L_{f}$ is the final height of the sample.

**Figure 3:**
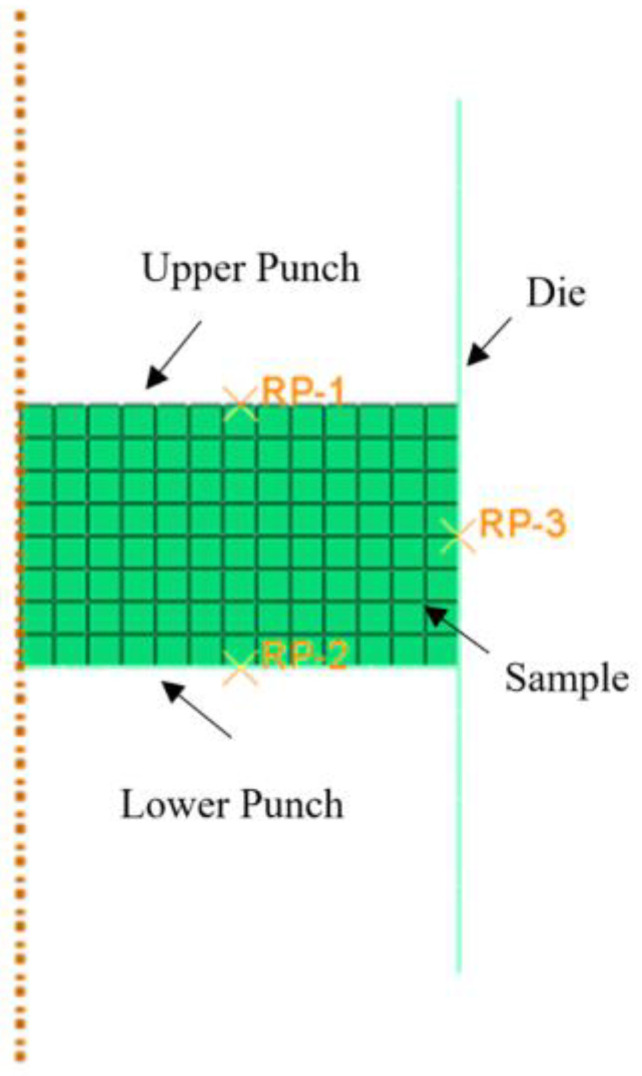
Axisymmetric FEM model.

The axisymmetric FEM model for the die compaction process is shown in Figure 3. The upper punch and die were fixed, while the lower punch was allowed to move forward according to the actual pressure-time curve. The displacement-time curve of the lower punch was fitted to the experimental data as simulation data. The parameters of the DPC model were set as input values, while the difference between the experimental and simulated displacement-time curves were set as the objective function, which can be expressed as:

(9) $$\varphi =\frac{1}{n}\sqrt{\sum_{i=1}^{n}{\left(D_{i}^{cal}-D_{i}^{exp}\right)}^{2}}$$

**Figure 4:**
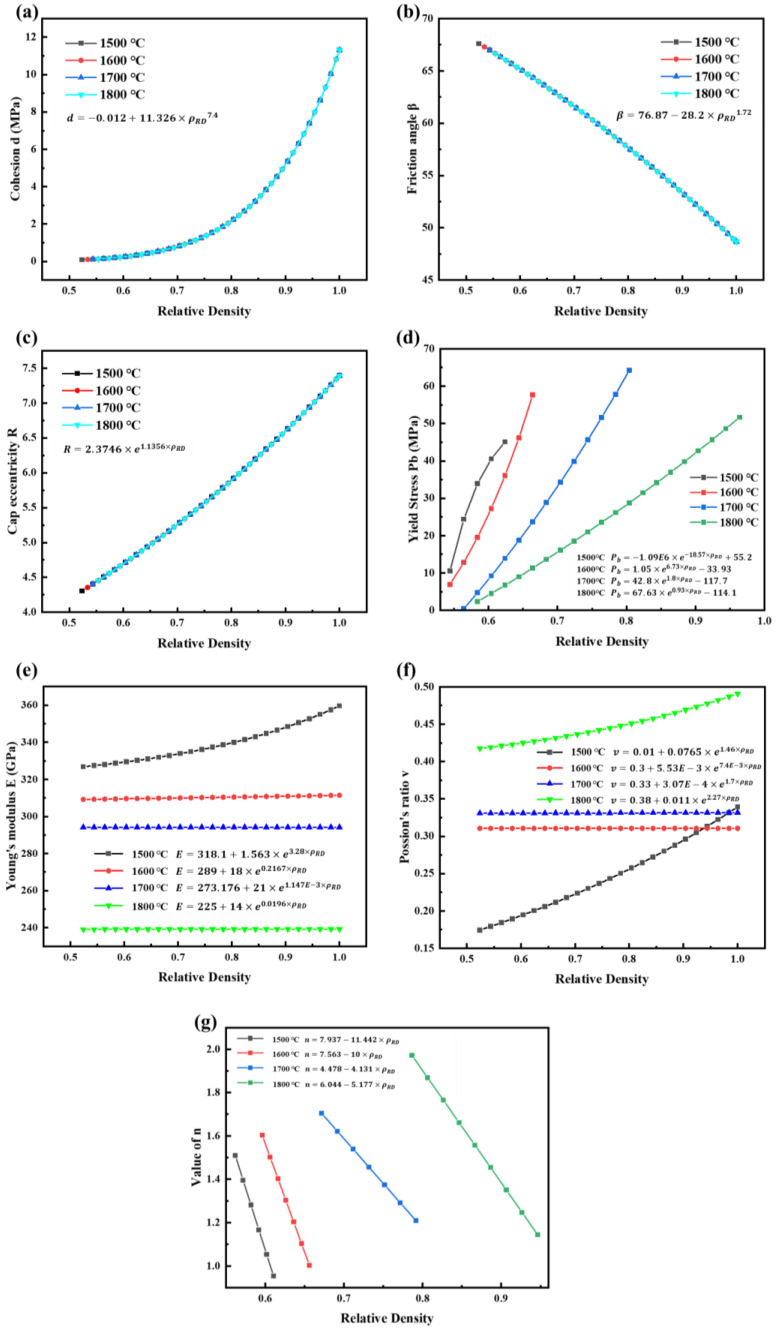
The elastic parameters and DPC parameters for B_4_C: (**a**) d, (**b**) β, (**c**) R, (**d**) P_b_, (**e**) E, (**f**) v, and (**g**) n. The variable “$\rho_{RD}$” represents relative density in the formulas.

**Figure 5:**
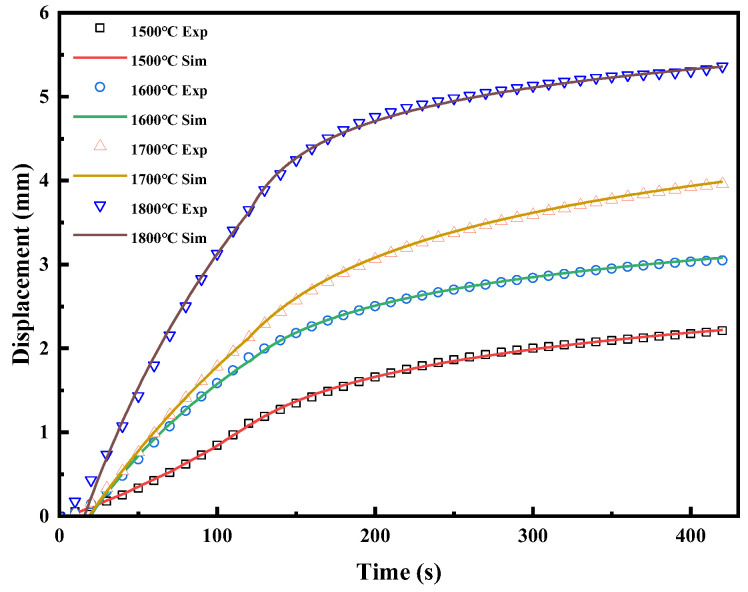
Experimental and simulated displacement-time curves for different temperatures.

The simplex method was used to take the minimum value of the objective function as the optimization objective [37]. This was implemented in the software MODEFRONTIER. Finally, the parameters of the DPC model and the elastic part were determined for B_4_C powder at 1500 °C, 1600 °C, 1700 °C, and 1800 °C, as shown in Figure 4. In addition, $A=10^{-5}$ and m=−0.1 were taken for the model. The experimental and simulated displacement-time curves are shown in Figure 5. At each temperature, the experimental and simulated curves fit almost perfectly. The errors were 0.19%, 0.40%, 0.42%, and 0.85%, respectively.

### 2.3. Finite Element Modeling

**Figure 6:**
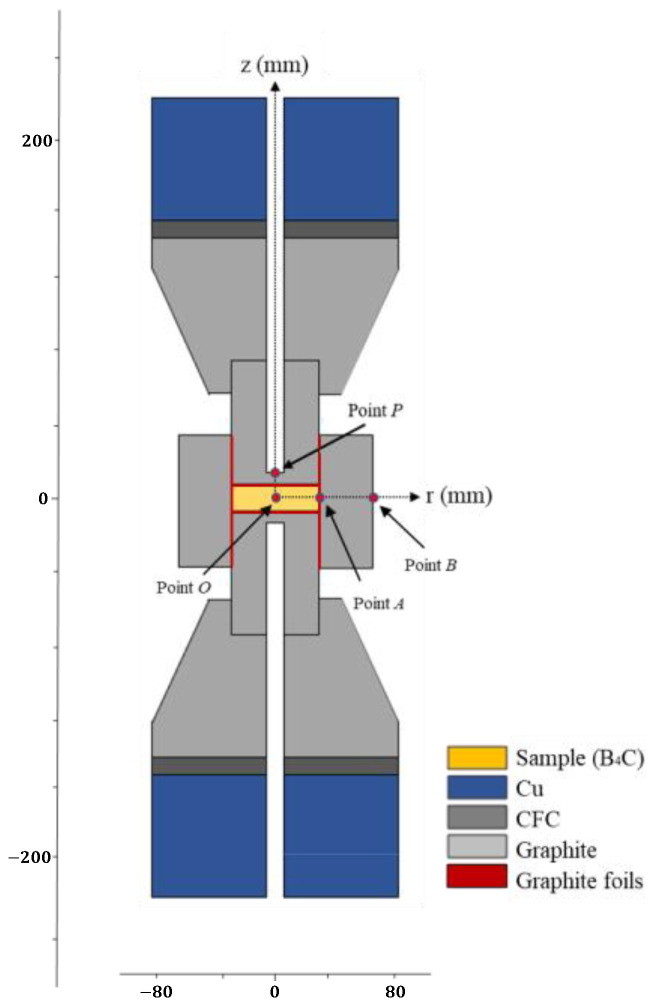
Schematic of the FCT system.

A finite element model was established to analyze the sintering process of boron carbide at 1800 °C. The geometry of the FCT system is shown in Figure 6. It includes two Cu electrodes, two carbon fiber composites (CFCs), two graphite spacers, two graphite punches, a graphite die graphite foil, and B_4_C sample. Point P is the location of the pyrometer, which is 3 mm above the sample.

The thermo-electric part was based on the equation of energy conservation (Abaqus^®^: Theory Manual and analysis user’s Manual):

(10) $$∰\rho C_{p}\frac{\partial \theta }{\partial t}dV=∰\nabla \left(k\nabla \theta \right)dV+∰{\dot{q}}_{e}dV+∯({\dot{q}}_{c}+{\dot{q}}_{conv}+{\dot{q}}_{r}+{\dot{q}}_{ec})dS$$

where ρ is the density of the material, $C_{p}$ is the specific heat capacity, θ is the temperature, k is the thermal conductivity, and t is the time. ${\dot{q}}_{e}$ represents the heat generated through Joule heat in volume V. ${\dot{q}}_{c},{\dot{q}}_{conv},{\dot{q}}_{r},and{\dot{q}}_{ec}$ represent the adjacent volume heat conduction, convective heat transfer, radiative heat transfer, and surface heat flux due to interfacial heat effects. ${\dot{q}}_{conv}=0$ since our experiment was conducted in vacuum conditions. The heat exchange was calculated using the following equations:

(11) $${\dot{q}}_{e}=\left(\nabla \varphi \right)\cdot \sigma (\nabla \varphi )$$

(12) $${\dot{q}}_{c}=h_{g}(\theta_{1}-\theta_{2})$$

(13) $${\dot{q}}_{r}=\sigma_{s}\varepsilon \left[{\left(\theta_{1}\right)}^{4}-{\left(\theta_{2}\right)}^{4}\right]$$

(14) $${\dot{q}}_{ec}=J\left(\varphi_{1}-\varphi_{2}\right)=\sigma_{g}{\left(\varphi_{1}-\varphi_{2}\right)}^{2}$$

where σ is the conductivity, φ is the electric potential, and $h_{g}$ and $\sigma_{g}$ are the gap thermal conductivity and gap electrical conductivity, respectively. $h_{g}=2\times 10^{5}\;WM^{-2}K^{-1}$ and $\sigma_{g}=3.2\times 10^{7}\;\Omega^{-2}M^{-1}$ was taken for the model [28]. $\sigma_{s}$ is the Stefan-Boltzmann constant, and ε is the emissivity (which is taken as 0.8) [29].

The mechanical property of the B_4_C powder was described using the DPC model, which was considered as a bivariate function of temperature and relative density. The process to obtain the parameters of the DPC model is described in Section 2.2. The thermal and electric properties of the powder and the thermal, electric, and mechanical properties of the other components were taken from Ref [28].

The boundary and initial conditions were set as follows:

- A predefined temperature field of 300 K was applied to the entire system; the upper and lower surfaces of the electrodes were set to a constant temperature of 300 K.
- The potential on the lower surface of the electrode was 0, and the actual current-time curve was applied on the upper surface.
- The upper surface of the electrode was fixed, and the actual displacement-time curve was applied to the lower surface of the electrode.
- Assuming a uniform distribution of the relative density of the material before sintering, a predefined relative density field was applied to the powder with a value of 0.58.

**Figure 7:**
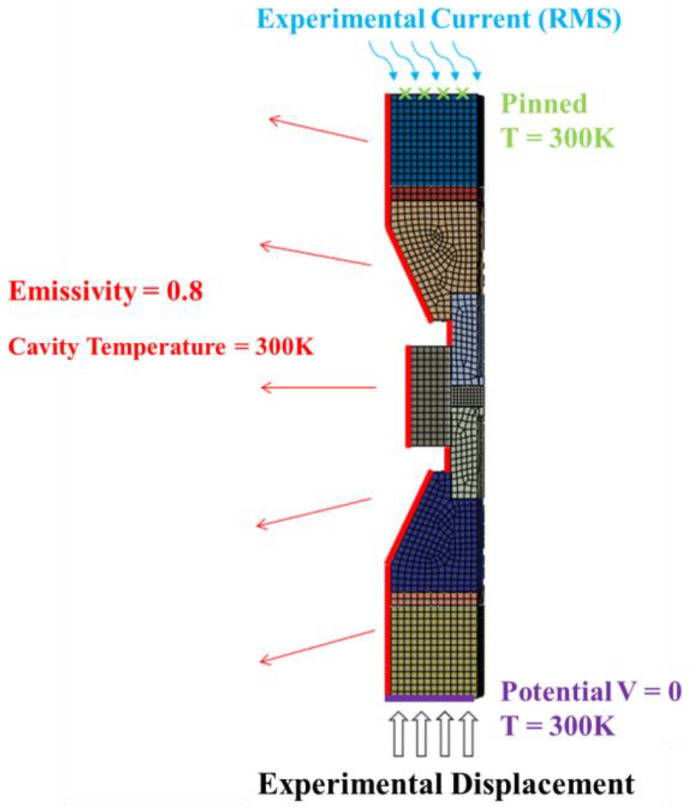
Boundary and initial conditions of the simulated SPS system.

The schematic of boundary and initial conditions is shown in Figure 7.

## 3. Results

### 3.1. Characterization of Raw Powder

**Figure 8:**
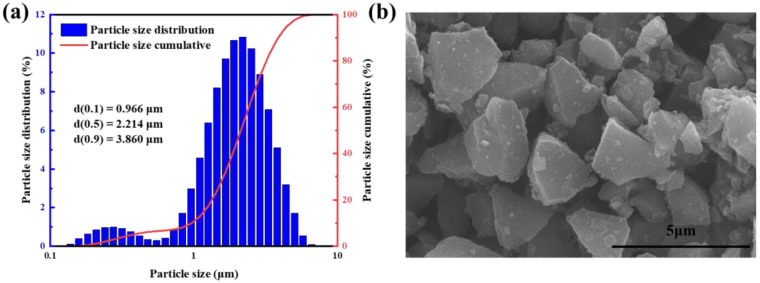
(**a**) Particle size distribution, (**b**) SEM image showing powder morphology.

The particle distribution and SEM image of B_4_C powder are shown in Figure 8. The average particle size of the powder was 2 μm, and it had good dispersion.

### 3.2. Electrical and Thermal Fields

**Figure 9:**
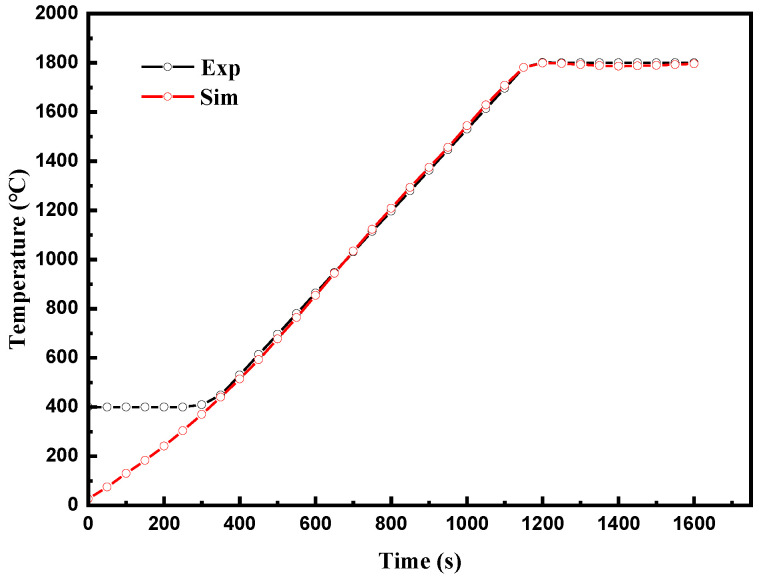
Comparison of simulation and experimental curves at temperature measuring points.

The temperature change was recorded by the pyrometer at point P (as shown in Figure 7); this is compared with simulated temperatures in Figure 9. As can be seen, there is a good agreement between experimental data and simulated data. It is worth noting that the pyrometer operates above 400 °C, so the experimental data are a horizontal line below 400 °C. Formula (8) was used to quantify the error between the experimental and simulated temperature-time curves, and only the part above 400 °C was considered. The calculated error value was 2.33%. The numerical model can well-simulate the thermal performance of the system, so the model can be further used to simulate the current density field and temperature field.

**Figure 10:**
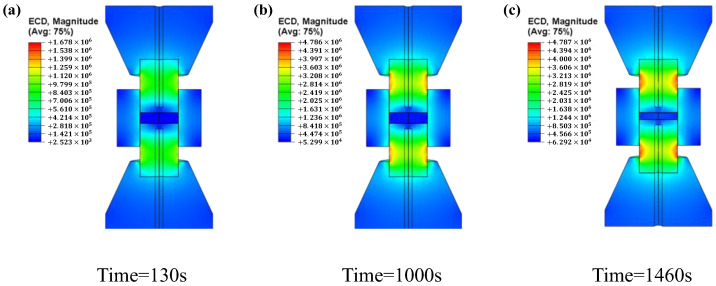
Current density versus time: (**a**) t = 130 s, (**b**) t = 1000 s, and (**c**) t = 1460 s.

Figure 10 shows the current density distribution of the system versus time, where Time = 300 s and Time = 1000 s are the current density distributions during the heating up process and Time = 1460 s is the current density distribution when the system reaches a steady state. It can be seen that the higher current density is always located at the punch/die interface and the punch/spacer interface, which are related to the sharp reduction in the geometric cross section. 

**Figure 11:**
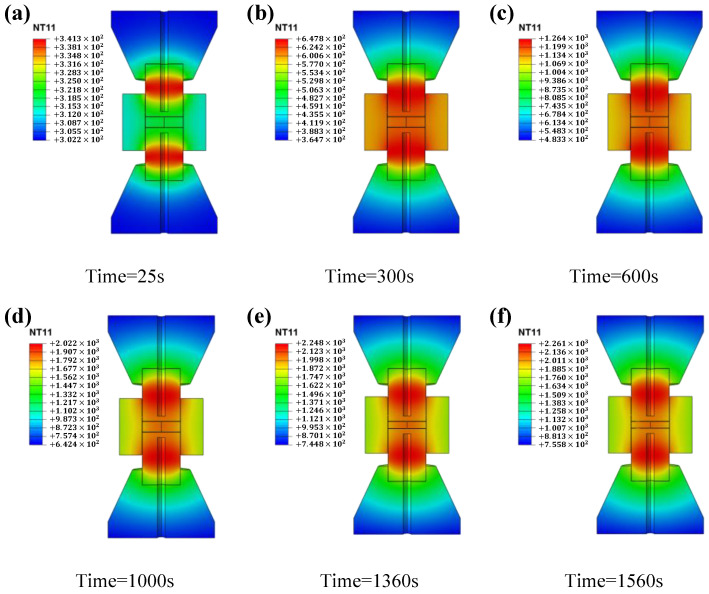
Temperature distribution versus time: (**a**) t = 25 s, (**b**) t = 300 s, (**c**) t = 600 s, (**d**) t = 1000 s, (**e**) t = 1360 s, (**f**) t = 1560 s.

The temperature distribution of the system versus time during sintering is shown in Figure 11. In agreement with the previous analysis, the temperature of the upper and lower punch is the highest due to the highest current density at the punch/die and punch/spacer interfaces, which serve as the main heating source for the sample. As the sintering proceeds, a radial temperature gradient starts to appear at the center surface of the sample, which is caused by the thermal radiation between the die and the external environment.

**Figure 12:**
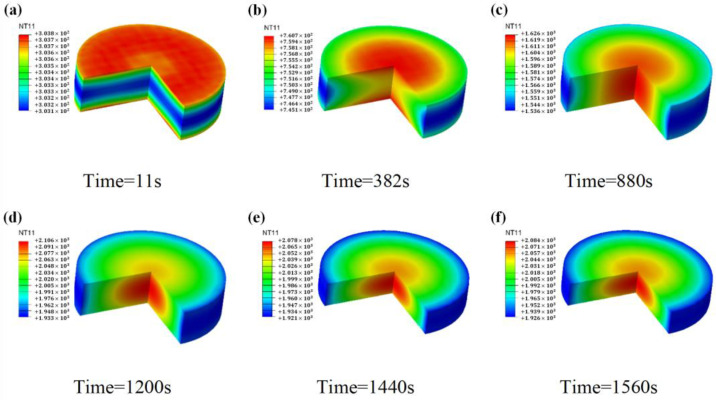
Temperature distribution of boron carbide sample versus time: (**a**) t = 11 s, (**b**) t = 382 s, (**c**) t = 880 s, (**d**) t = 1200 s, (**e**) t = 1440 s, (**f**) t = 1560 s.

Figure 12 shows the variation pattern of temperature versus time inside the B_4_C sample. The highest temperature appears first on the top and bottom surfaces, and then moves toward the center of the sample. As the sintering temperature increases, the electrical and thermal conductivity of the sample start to rise and spontaneous Joule heating starts to be generated inside the sample. A steady-state temperature distribution is formed inside the sample during the soaking time. During the pressurization process, the temperature distribution trend remains the same, although the temperature magnitude changes slightly.

**Figure 13:**
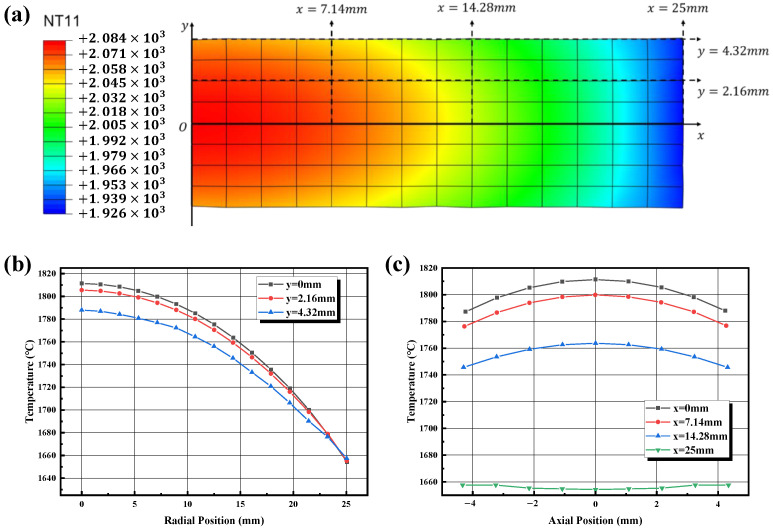
(**a**) Temperature distribution of sample profile, (**b**) radial temperature gradients at different locations, (**c**) axial temperature gradients at different locations.

Figure 13 shows the longitudinal profile of the boron carbide sample during the soaking time; a two-dimensional coordinate system was introduced to quantitatively describe its temperature distribution. The radial temperature difference is much larger than the axial temperature difference. The radial temperature difference on the middle surface is the largest, reaching 150 °C, while the maximum temperature difference in the axial direction is only 25 °C.

### 3.3. Stress Fields

**Figure 14:**
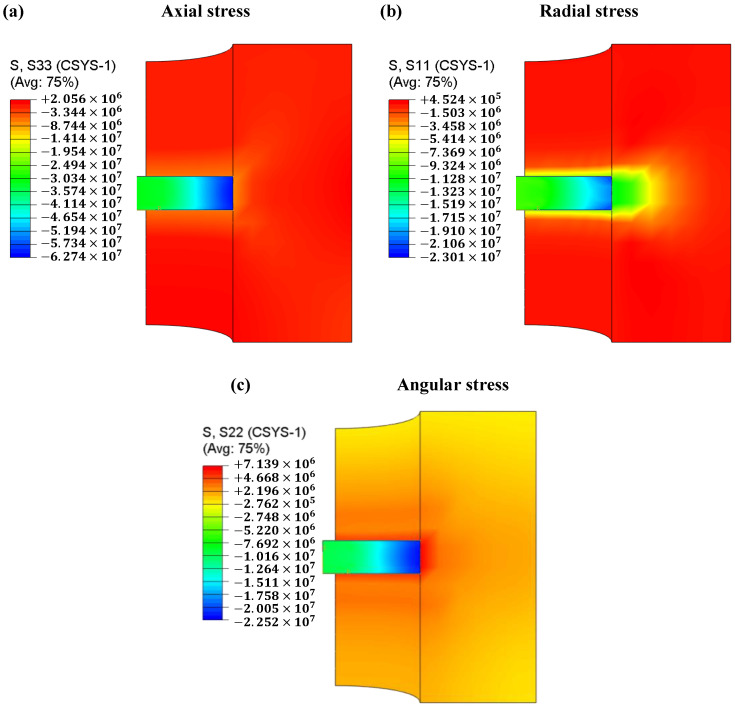
Contour plots of stresses in the (**a**) axial, (**b**) radial, and (**c**) angular directions.

**Figure 15:**
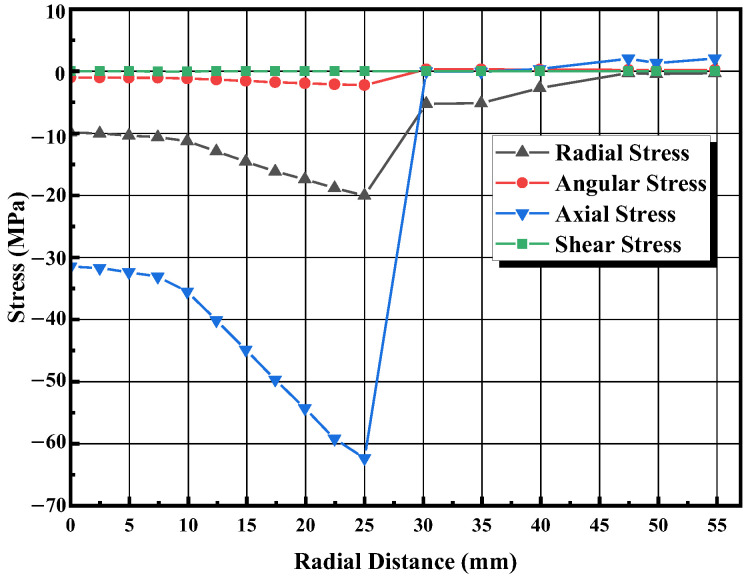
Distributions of axial, radial, angular, and shear stresses.

As Figure 14a shows, there is a relatively large axial stress gradient in the samples because of the different temperatures and sintering properties in different regions. The axial stress in the die is very small, probably because the introduction of graphite foils reduces the mechanical friction between the powder and the die. Figure 14b shows the contours of radial stress distribution. In the radial direction, both the sample and the die are in a compressed state. The sample is subjected to a much higher compressive stress than the die. The compressive stress is also well-distributed throughout the graphite die, except for the area in contact with the powder, and there is no stress on the outer surface of the die. Figure 14c shows the contours of angular stress distribution. The angular stresses change from compression in the sample to tension in the die. Figure 15 shows the specific values of stress distribution along the diameter through the center of the sample. Considering that the thermal expansion coefficient of boron carbide (5.73 × 10^−6^ K^−1^) is smaller than that of the graphite mold (8 × 10^−6^ K^−1^), the interaction between the two caused by thermal expansion is negligible. Therefore, the radial and angular stresses generated by the graphite mold are mainly from the Poisson expansion of the powder.

### 3.4. Relative Density Fields

**Figure 16:**
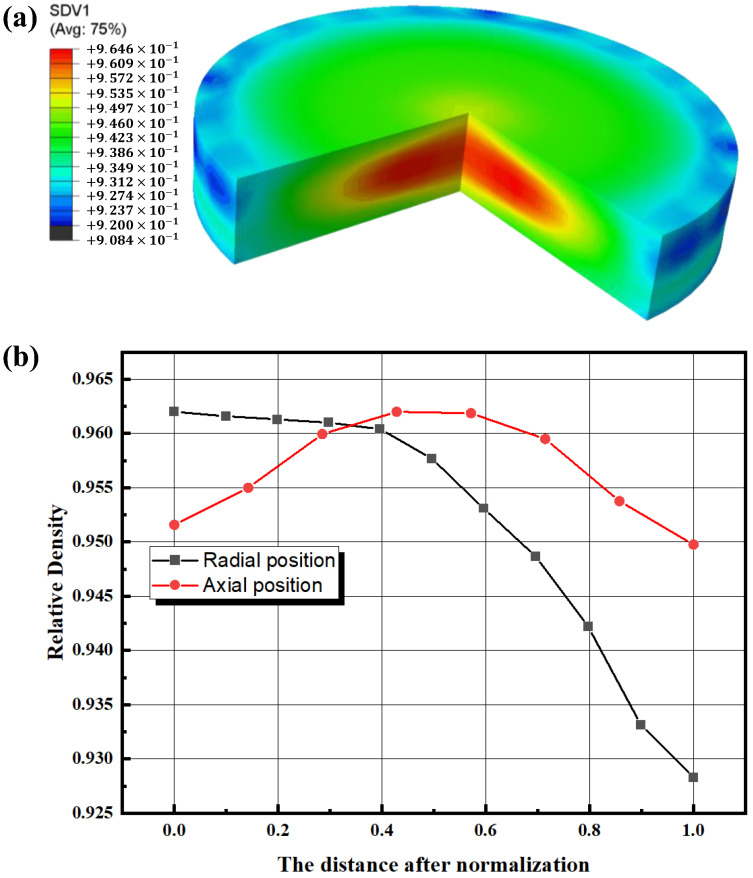
(**a**) Contour map of relative density of the sample, (**b**) axial and radial relative density gradient values of the sample.

The relative density distribution inside the sample is shown in Figure 16. From Figure 16a, it can be seen that there is an ellipsoidal region with the highest densities inside the sample, and the relative density gradually decreases along the ellipsoidal region outward. Figure 16b shows the axial and radial relative density gradients along the sample center; the axial relative density gradient is relatively limited, the maximum value appears at the center with a value of 0.962, the relative density decreases from the center to both sides, and the relative density value of the upper and lower surfaces is 0.954. The relative density gradient in the radial direction is larger, the maximum value is still at the sample center, and the relative density decreases gradually along the radius. This is because, on the one hand, the temperature in this region is the lowest and the sintering performance of the powder is poor. On the other hand, there is mechanical friction between the powder and the graphite foils, which reduces the mobility of the powder, so the relative density value at the edge of the powder is small. The trend of the relative density distribution is in good agreement with the distribution of the temperature field because the parameters of the DPC model of the powder were set as a function of temperature, so different temperature regions have different sintering properties.

**Figure 17:**
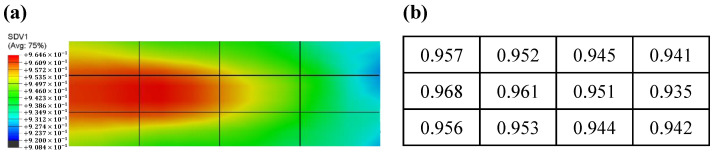
(**a**) Simulated relative density, (**b**) experimental relative density.

The sample was cut into 3 × 4 pieces for comparison with the simulation results, and the cutting diagram is shown in Figure 17. The bulk relative densities of different regions of the powder were determined using the Archimedes’ method. The results show good agreement between the simulated and experimental relative density distributions. To quantify the agreement, the Pearson product-moment correlation coefficient formula [38] was used to calculate the Pearson correlation coefficient between the simulated and experimental relative density fields. The simulated relative density matrix is *X* = [0.956, 0.954, 0.949, 0.941, 0.963, 0.961, 0.955, 0.937, 0.956, 0.953, 0.948, 0.943], while the experimental relative density matrix is *Y* = [0.957, 0.952, 0.945, 0.941, 0.968, 0.961, 0.951, 0.935, 0.956, 0.953, 0.944, 0.942], respectively. The final calculated Pearson correlation coefficient value is 0.9699, indicating a strong correlation between the simulated and experimental values, which proves the correctness of the model.

## 4. Discussion

**Figure 18:**
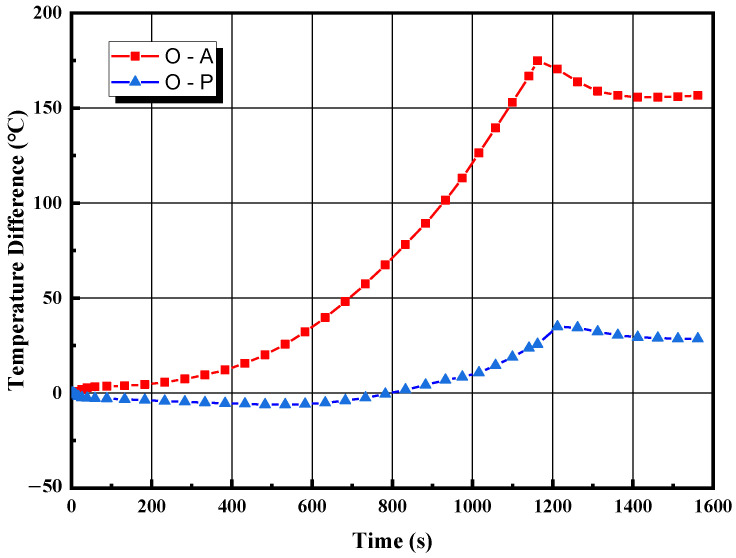
The difference in temperature between different positions over time.

The densification mechanism of SPS is still controversial. Some scholars attribute the ability of SPS to lower sintering temperature to the inaccuracy of temperature measurements at the measuring points. In existing SPS equipment, two positions, such as points A and P shown in Figure 6, are commonly used as infrared temperature measurement points. The temperature differences between the sample center and point A, as well as between the sample center and point P, are plotted in Figure 18. When the system reaches thermal steady-state, the temperature difference between points O and P is around 25 °C, while the temperature difference between points O and A is as high as 150 °C.

This explains why there is a significant difference in the performance of sintered samples when using different temperature measurement points at the same nominal temperature. In addition, for the sintering of boron carbide samples, it is recommended to select a position above the sample as the temperature measurement point, as it can better reflect the temperature of the sample center.

Choosing the appropriate mold size is crucial for the success of the sintering process, as it can impact the quality, performance, production efficiency, and cost of the product.

**Figure 19:**
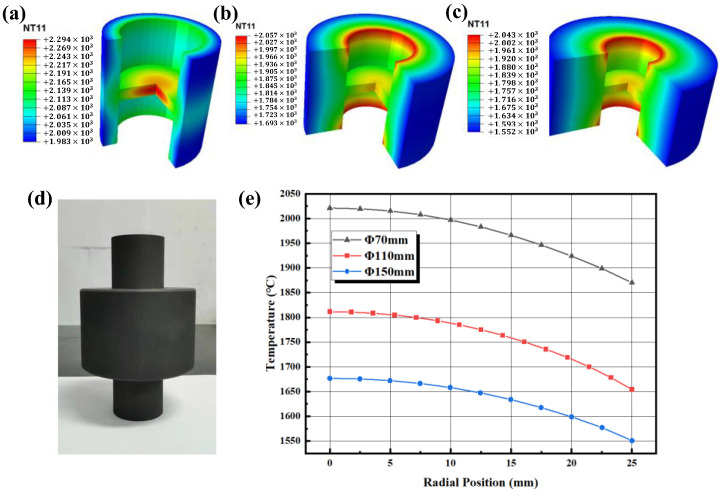
(**a**) Φ70 mm, (**b**) Φ110 mm, (**c**) Φ150 mm, (**d**) mold with Φ110 mm, (**e**) radial temperature of the sample at different diameters.

Three types of molds with sizes of Φ70 mm, Φ110 mm, and Φ150 mm were designed, and the temperature distribution of the final samples was calculated. Figure 19 shows the radial temperature distribution of the samples using molds of different sizes. The larger the mold diameter, the lower the temperature of the sample, indicating that increasing the diameter will reduce the thermal efficiency. The size of the temperature gradient was quantified using the following formula:

(15) $$\omega =\frac{T_{max}-T_{min}}{T_{min}}$$

where $T_{max}$ represents the maximum radial temperature and $T_{min}$ represents the minimum radial temperature. For the three types of molds with sizes of Φ70 mm, Φ110 mm, and Φ150 mm, the temperature gradients are 8.08%, 9.50%, and 8.13%, respectively. The influence of mold size on temperature gradient is not monotonic.

On the premise of ensuring that the mold does not crack or deform, it is advisable to use molds with the smallest possible diameter. On the one hand, this can improve the thermal efficiency of the system, saving costs; on the other hand, it can also reduce the axial temperature asymmetry of the samples caused by the gravity of the mold.

## 5. Conclusions and Outlook

A coupled thermal-electrical-mechanical finite element model was established to analyze the SPS process of boron carbide samples sintered at 1800 °C. The DPC model was employed to simulate the densification behavior during the sintering process, taking into account both the plastic deformation and creep mechanism within the DPC model. According to the simulation results, the following conclusions can be drawn:

- A radial temperature gradient of up to 155 °C was present in the sample when the thermal steady state was reached, while the axial temperature gradient was relatively limited, only up to 24 °C. The radial temperature generation could be attributed to the heat radiation loss from the die.
- According to the stress field analysis, a large axial stress gradient and a radial stress gradient existed inside the sample. The simulated relative density distribution field was compared with the experimentally measured values, and the Pearson correlation coefficient between the two distributions was 0.9699, confirming the validity of the model.
- When sintering boron carbide ceramics using the SPS device, selecting a temperature measurement point above the sample can better reflect the true temperature of the sample.
- Reducing the mold diameter can improve thermal efficiency, while not significantly affecting the thermal gradient.

In future work, further investigation will be conducted on the details of the model, such as the effect of grain size on the model parameters and the thermoelectric effect of boron carbide.

## Data Availability

Not applicable.
